# Development of Caregiving Typologies: Understanding Decision Making and Prioritization in Dementia Caregiving

**DOI:** 10.1093/geroni/igaf122.1643

**Published:** 2025-12-31

**Authors:** Henrietta Bennett, Vicki Winstead, Carolyn Pickering

**Affiliations:** University of Alabama at Birmingham, Birmingham, Alabama, United States; Cizik School of Nursing at UTHealth Houston Texas, Houston, Texas, United States; UTHealth Houston, Houston, Texas, United States

## Abstract

Family caregivers of individuals with dementia navigate complex and often competing care demands, requiring them to make difficult decisions about task prioritization. As the disease progresses and care needs intensify, balancing immediate concerns with long-term goals becomes increasingly challenging, both emotionally and practically. The study aimed to assess how caregivers prioritize care tasks through typologies of caregiving to understand their decision-making processes and approach to caregiving. 20 family caregivers who lived with and provided unpaid care to a family member living with dementia were purposefully selected from a national study on caregiver decision-making. Semi-structured interviews were conducted to explore how caregivers prioritized and navigated decisions regarding daily care tasks. Using a grounded theory approach, two qualitative coders coded and compared codes at each stage of coding and collapsed codes into thematic categories of caregiving. We identified two primary caregiving typologies: Proactive and Reactive, based on whether caregivers plan or initiate decisions in arising care situations. The proactive-reactive typology captures the heterogeneity of caregiving experiences and provides better understanding of the mechanisms behind caregiver decision-making process. The study revealed that both typologies are influenced by multiple individual, interpersonal and structural factors including caregiver-care recipient relationship type and quality, caregiving values, and support. Findings from the study provides a useful framework for understanding family caregivers decision-making processes and care prioritization. Given the dynamic and context-dependent nature of daily caregiving, targeted interventions should consider the variability between these typologies, ensuring that caregivers are equipped with resources to mitigate stressors associated with crisis-driven decision-making.

